# Supplementary figures and images for: RAMP1 in Kupffer cells is a critical regulator in immune-mediated hepatitis
Source: PLoS One. 2018 Nov 21;13(11):e0200432. doi: 10.1371/journal.pone.0200432 (PMC6248891; doi:10.1371/journal.pone.0200432)

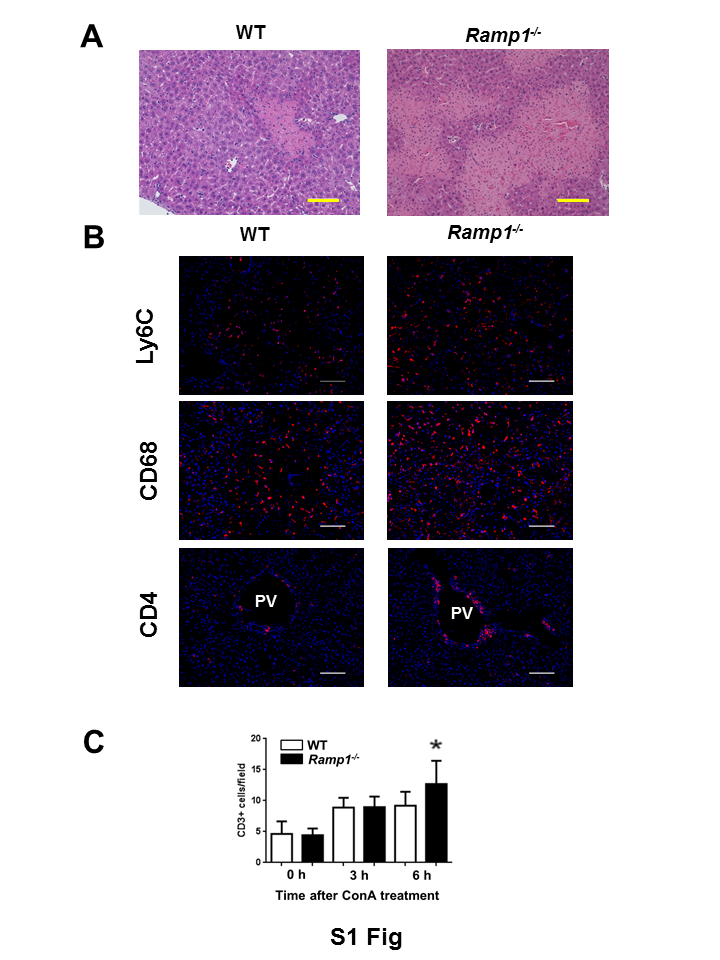

Supplement: S1 Fig — (TIF) [file pone.0200432.s001.TIF]

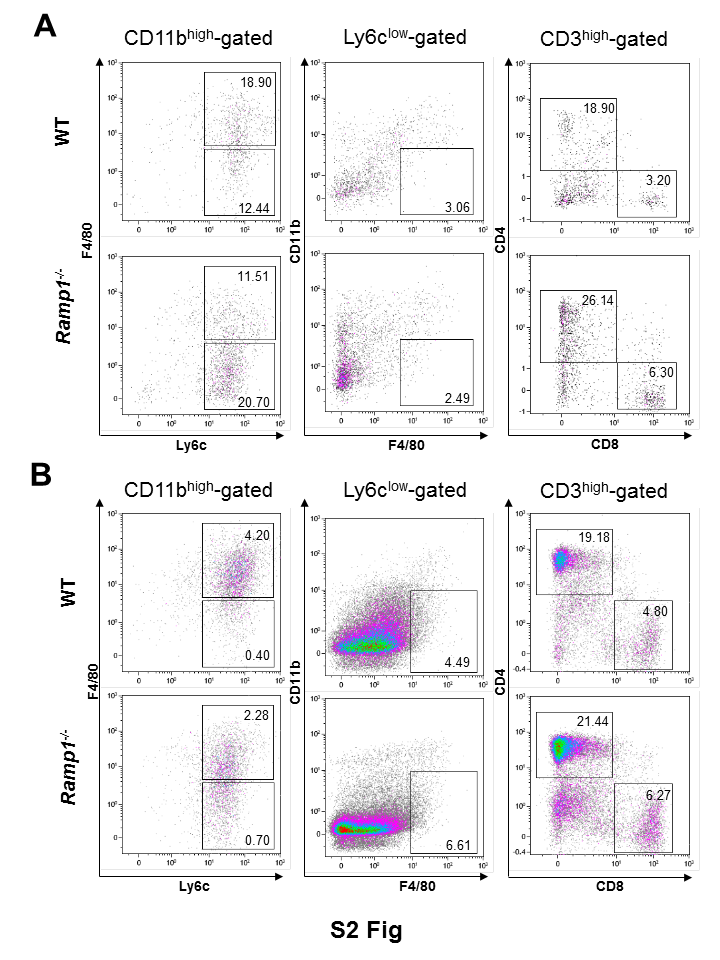

Supplement: S2 Fig — (TIF) [file pone.0200432.s002.TIF]

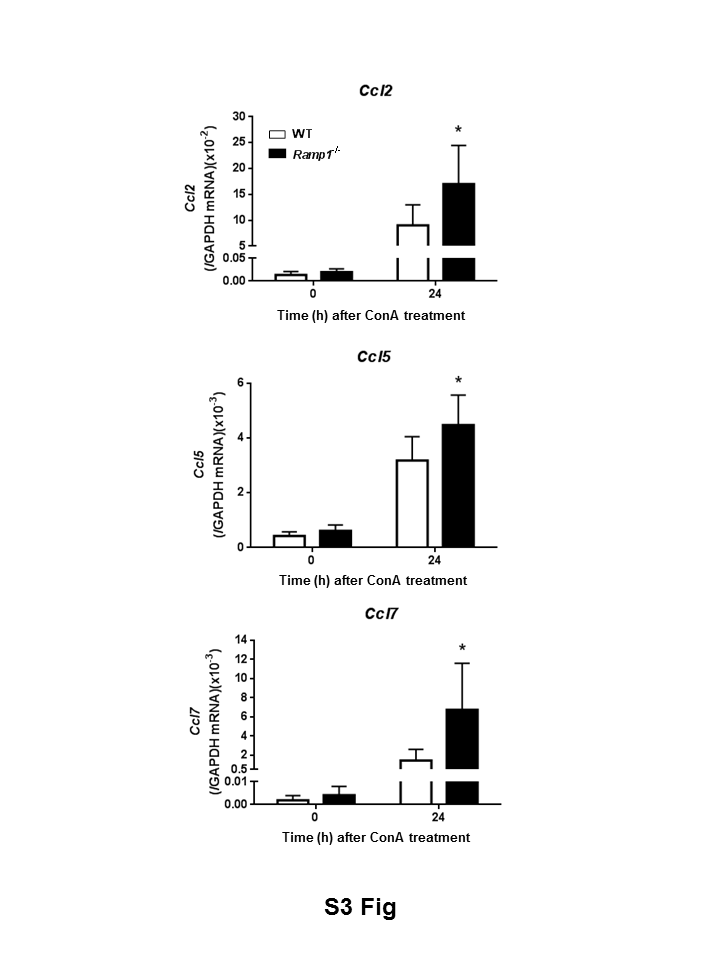

Supplement: S3 Fig — (TIF) [file pone.0200432.s003.TIF]

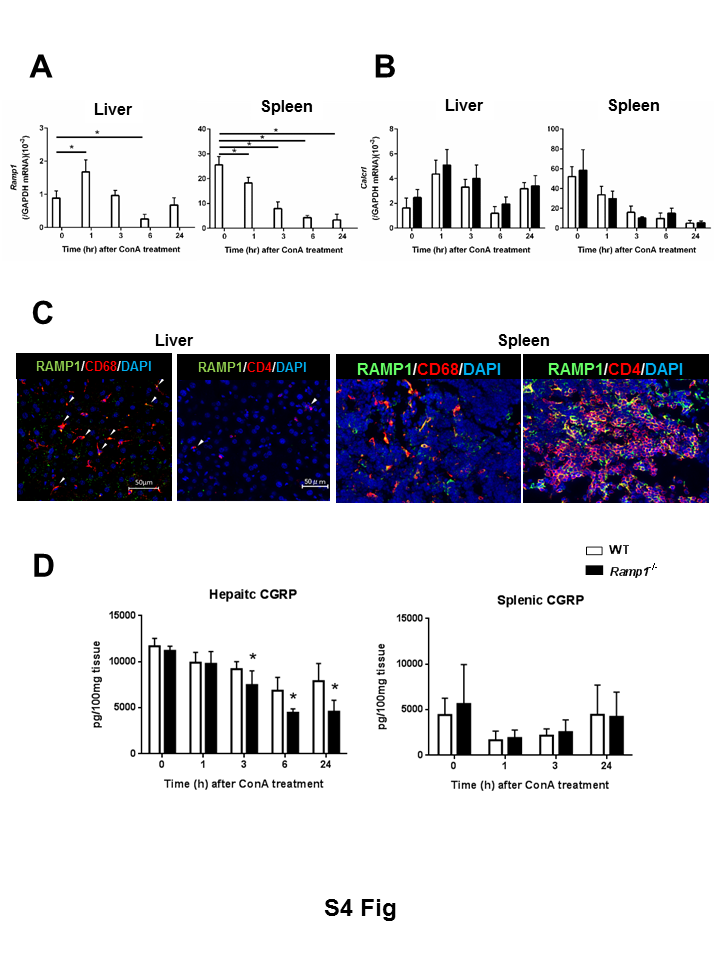

Supplement: S4 Fig — (TIF) [file pone.0200432.s004.TIF]

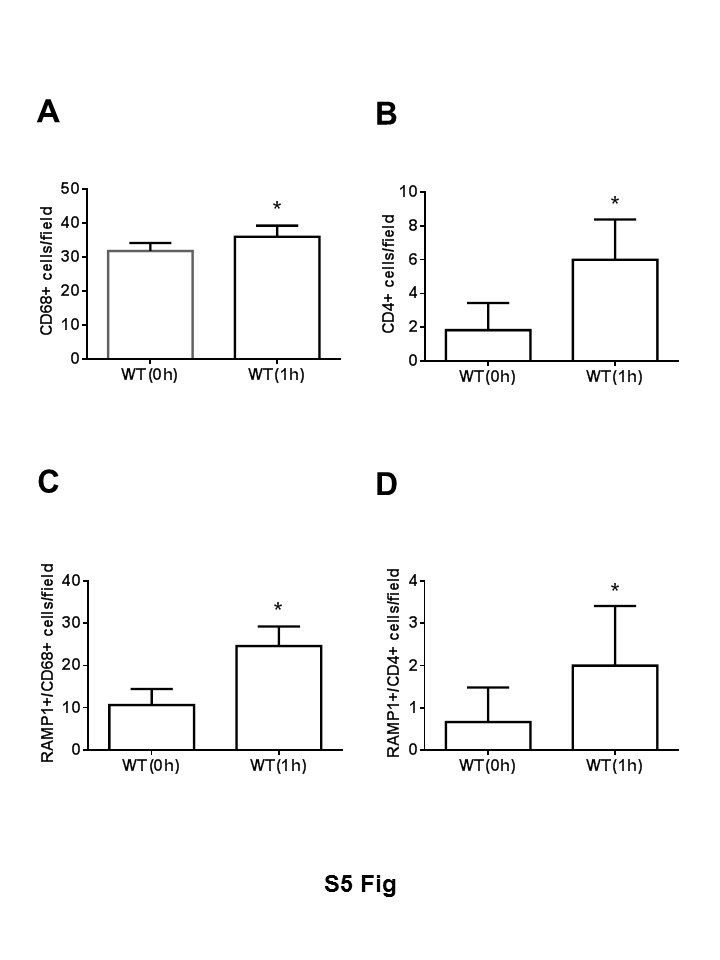

Supplement: S5 Fig — (TIF) [file pone.0200432.s005.TIF]

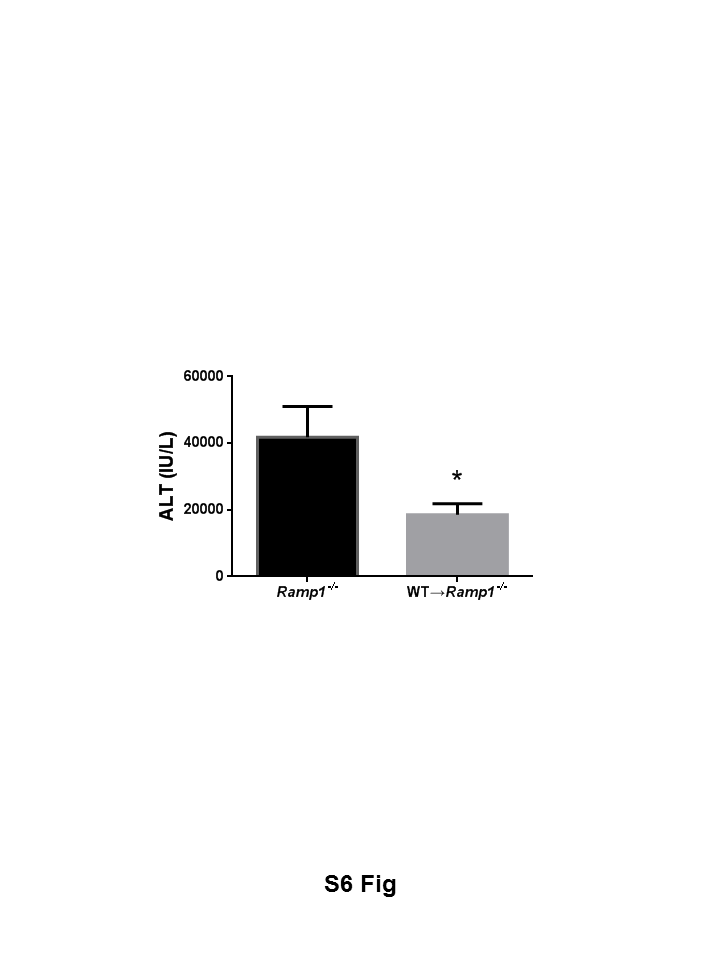

Supplement: S6 Fig — (TIF) [file pone.0200432.s006.TIF]

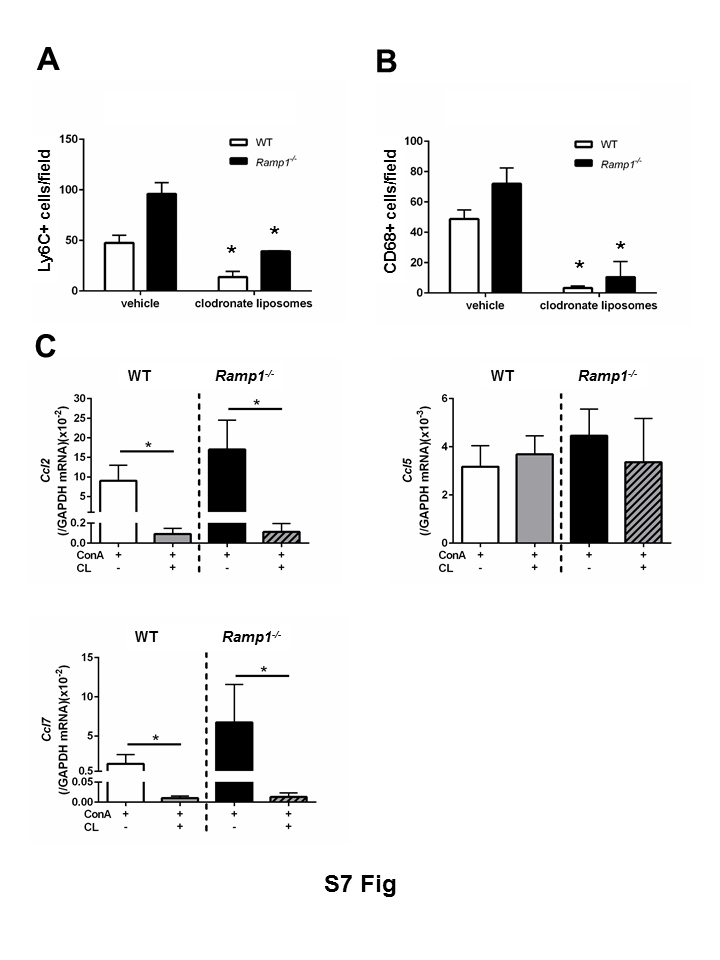

Supplement: S7 Fig — (TIF) [file pone.0200432.s007.TIF]

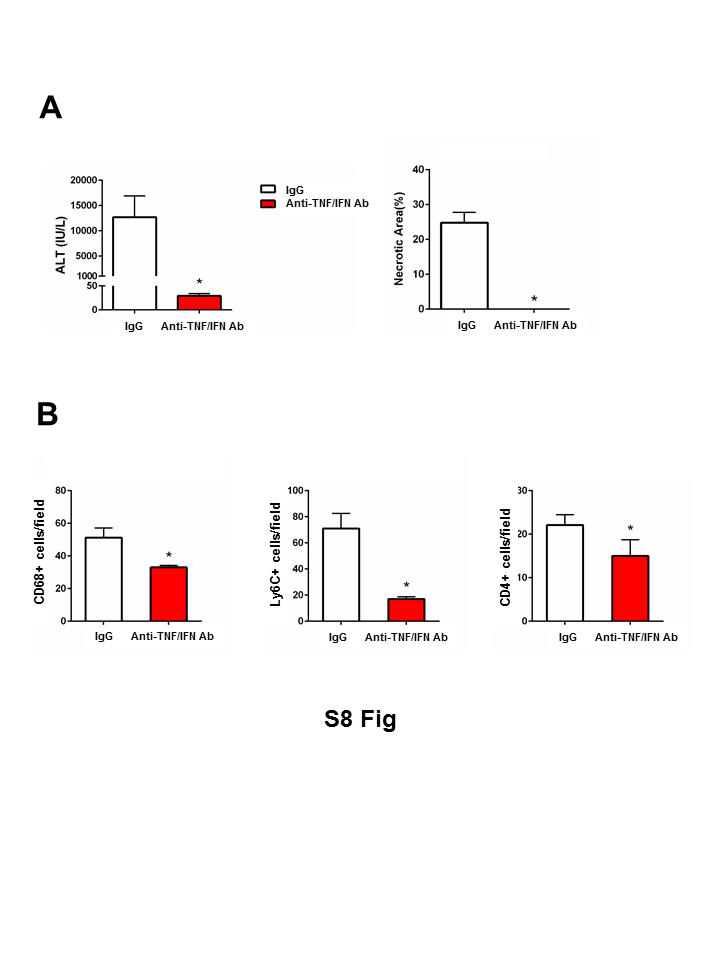

Supplement: S8 Fig — (TIF) [file pone.0200432.s008.TIF]

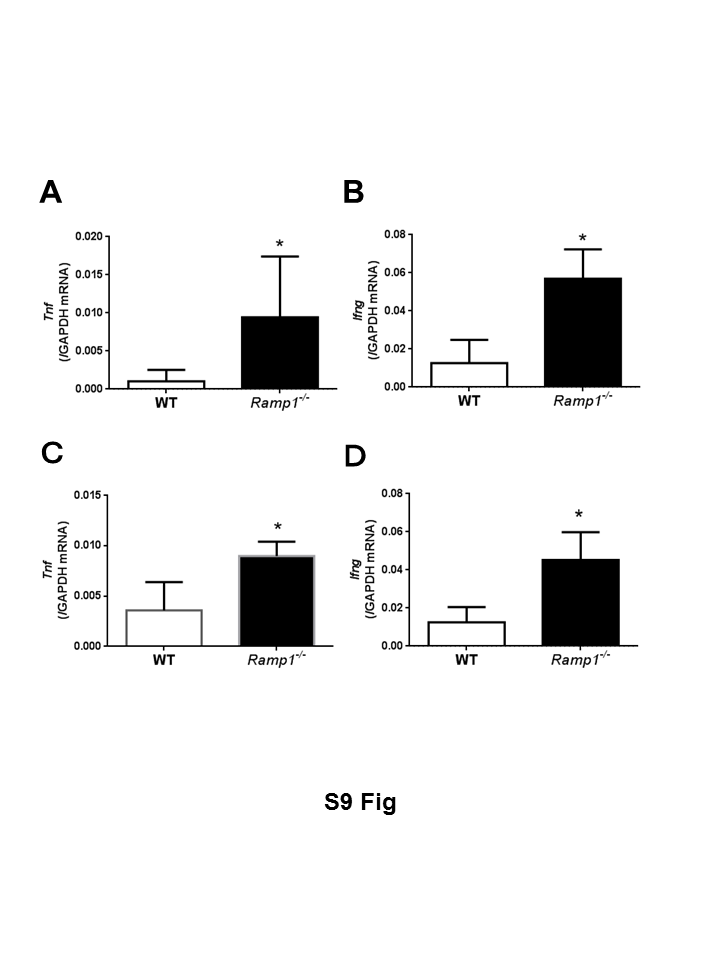

Supplement: S9 Fig — (TIF) [file pone.0200432.s009.TIF]

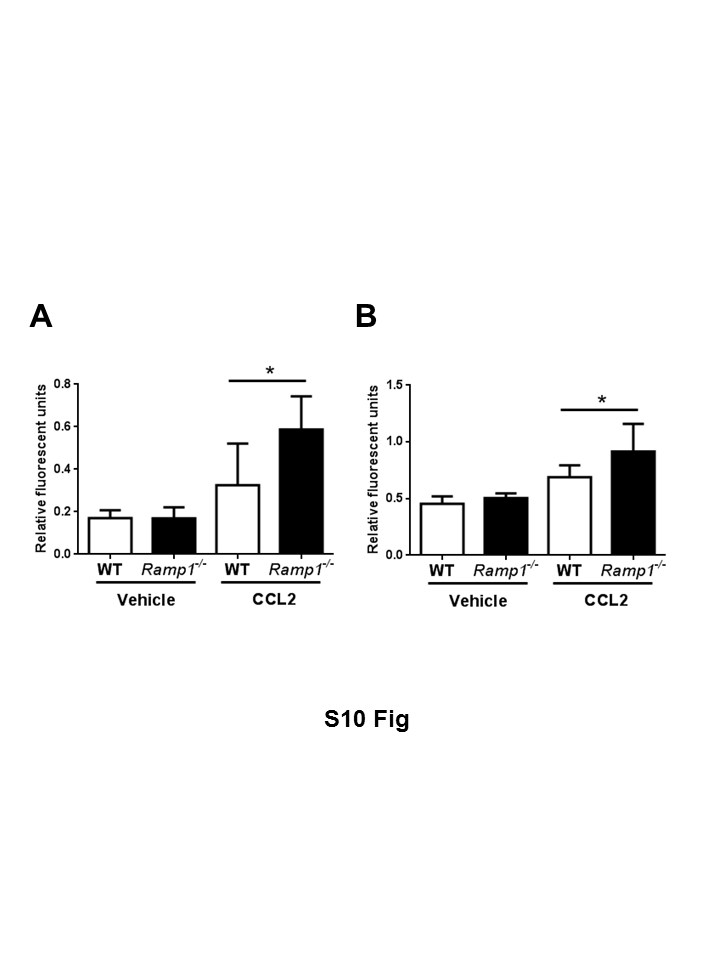

Supplement: S10 Fig — (TIF) [file pone.0200432.s010.TIF]

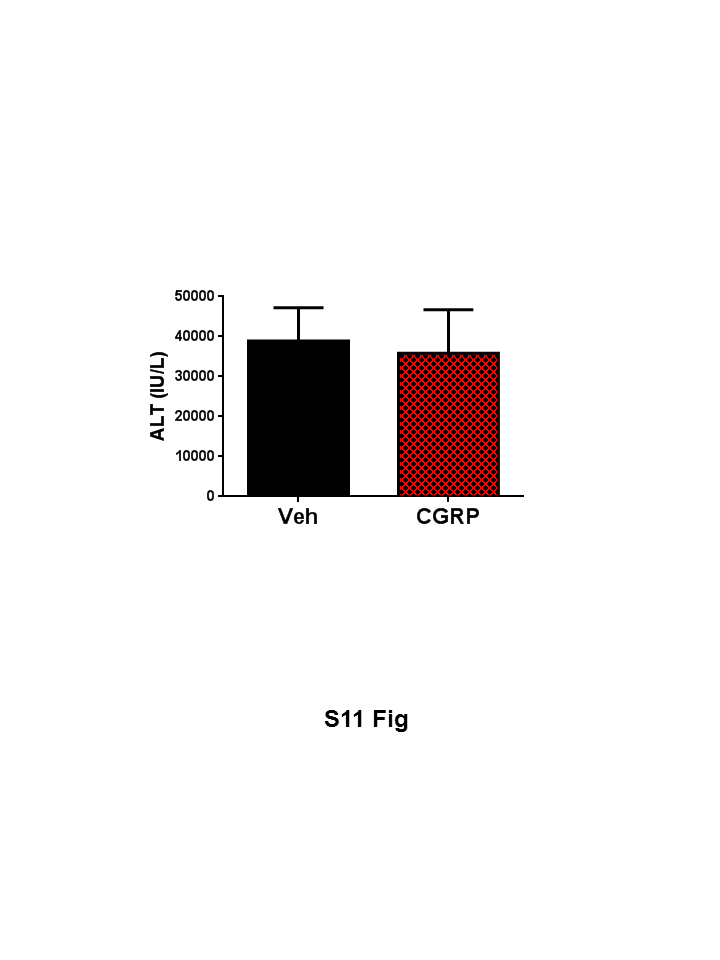

Supplement: S11 Fig — (TIF) [file pone.0200432.s011.TIF]
